# The Vascular Effects of Isolated Isoflavones—A Focus on the Determinants of Blood Pressure Regulation

**DOI:** 10.3390/biology10010049

**Published:** 2021-01-12

**Authors:** Henrique Silva

**Affiliations:** 1 Informetrics Research Group, Ton Duc Thang University, Ho Chi Minh City 758307, Vietnam; henriquesilva@tdtu.edu.vn; 2 Faculty of Pharmacy, Ton Duc Thang University, Ho Chi Minh City 758307, Vietnam

**Keywords:** isoflavones, vasorelaxation, endothelium, ion channels, estrogen receptor, tyrosine kinase

## Abstract

**Simple Summary:**

Isoflavones are naturally-occurring phytoestrogens, highly prevalent in soybeans, and known to improve cardiovascular health in populations with a high isoflavone dietary intake. Most clinical studies have assessed the impact of dietary intake or supplementation with mixtures of isoflavones, with few studies dedicated to the effects of isolated compounds (i.e., genistein, daidzein, glycitein, formononetin, biochanin A, and equol). This paper reviews the main actions of isolated isoflavones on the vasculature, with particular focus on the determinants of blood pressure regulation. Isoflavones evoke relaxation of different vascular beds by acting on several signaling pathways in the endothelium, where they potentiate the release of important vasorelaxant mediators, and in vascular smooth muscle cells, where relaxation is attained mainly through hyperpolarization. Some of these effects are attributed to their ability to modulate estrogen receptors. These vascular effects occur at plasma concentrations in the micromolar range, attained only through dietary supplementation. This paper highlights isolated isoflavones as potentially suitable alternatives to soy-based foodstuffs and supplements and which could enlarge the current therapeutic arsenal.

**Abstract:**

Isoflavones are phytoestrogen compounds with important biological activities, including improvement of cardiovascular health. This activity is most evident in populations with a high isoflavone dietary intake, essentially from soybean-based products. The major isoflavones known to display the most important cardiovascular effects are genistein, daidzein, glycitein, formononetin, and biochanin A, although the closely related metabolite equol is also relevant. Most clinical studies have been focused on the impact of dietary intake or supplementation with mixtures of compounds, with only a few addressing the effect of isolated compounds. This paper reviews the main actions of isolated isoflavones on the vasculature, with particular focus given to their effect on the determinants of blood pressure regulation. Isoflavones exert vasorelaxation due to a multitude of pathways in different vascular beds. They can act in the endothelium to potentiate the release of NO and endothelium-derived hyperpolarization factors. In the vascular smooth muscle, isoflavones modulate calcium and potassium channels, leading to hyperpolarization and relaxation. Some of these effects are influenced by the binding of isoflavones to estrogen receptors and to the inhibition of specific kinase enzymes. The vasorelaxation effects of isoflavones are mostly obtained with plasma concentrations in the micromolar range, which are only attained through supplementation. This paper highlights isolated isoflavones as potentially suitable alternatives to soy-based foodstuffs and supplements and which could enlarge the current therapeutic arsenal. Nonetheless, more studies are needed to better establish their safety profile and elect the most useful applications.

## 1. Introduction

Phytoestrogens are naturally occurring plant phenolic compounds resembling the molecular structure of estrogens, in particular 17-β-estradiol. They can exert both estrogenic and/or anti-estrogenic effects by acting on the estrogen receptors, changing the levels of sex-hormone-binding globulin (SHBG) and changing the estrogen plasma concentration and excretion [1,2]. In plants, phytoestrogens do not function as hormones, but rather as phytoalexins—inducible secondary metabolites that accumulate during stress and microbe attacks [3]. These compounds exert antimicrobial (i.e., fungistatic, antibacterial, and antiviral) and antioxidant properties, besides preventing the development of plant tumors due to their anti-angiogenic activity [4]. Phytoestrogens can be subdivided into two groups: flavonoids, which include isoflavones, coumestans, and prenylflavonoids; and non-flavonoids, which comprise lignans [5]. Isoflavones are considered to be very potent phytoestrogens, even though they display several other biological actions, including protective effects on the metabolism [6] and in the musculoskeletal [7] and cardiovascular systems [8], and having anticancer properties [9].

Isoflavones decrease overall cardiovascular risk by exerting important anti-hypertensive and anti-atherosclerotic effects in vitro and in vivo, the latter both in animal models as well as in clinical studies [10]. Most human studies conducted thus far have mainly investigated the effects of isoflavone-rich diets [11,12], plant extracts [13], and mixtures of isoflavones [14], with only a few studies having focused on the effect of isolated isoflavones.

The primary dietary source of isoflavones are legumes of the Fabaceae family [15], namely, soybeans (*Glycine max*), soy-based products (i.e., miso, tempeh, and tofu), lupin (*Lupinus* spp.), fava beans (*Vicia faba*), kudzu (*Pueraria lobata*), psoralea (*Psoralea* spp.), and red clover (*Trifolium pratense*) [5,16,17]. In these sources, particularly in soybeans and soy-based products, the predominant isoflavones with beneficial properties for the cardiovascular system are genistein, daidzein, and glycitein [18,19], and through regular dietary intake they reach physiologically relevant levels in the bloodstream [20,21,22,23]. Other isoflavones currently recognized as beneficial for cardiovascular health are formononetin and biochanin A, present in red clover [17], soybeans [24,25], peanuts (*Arachis hypogaea*) [26], chickpeas (*Cicer arietinum*) [27], Indian rosewood (*Dalbergia sissoo*) [28], golden tree (*Cassia fistula*) [29], and alfalfa sprouts (*Medicago sativa*) [30], as well as in Huangqi (*Astragalus membranaceus*) [31], a herb used in traditional Chinese medicine [17]. The concentration of formononetin and biochanin A is sufficiently low in these sources to prevent reaching physiologically relevant levels through dietary intake. As such, these isoflavones are employed in studies mainly as extracts or as isolated compounds [13,32,33,34]. Some of these compounds are metabolized by mammals to equol, an isoflavan that is not naturally occurring and therefore not considered a phytoestrogen, despite also possessing important biological effects [35]. In plants, isoflavones are typically present in their glycosylated form, which includes β-glycosides (i.e., genistin, daidzin, glycitin, puerarin, ononin, sissotrin, etc.), malonyl glycosides, and acetyl glycosides. The glycoside forms of isoflavones are considered biologically inactive in animals, gaining activity only after they are hydrolyzed and their corresponding aglycone forms are released and made available for absorption [36].

The beneficial effects of isoflavones for cardiovascular health have become increasingly apparent in recent decades due to the popularity of soy-based foodstuffs in Western diets. In fact, soybeans have been raised to the rank of a functional food since the discovery of their biological potency [37]. So far, it has been demonstrated that isoflavones display important vasorelaxant [38], anti-angiogenic [39], antioxidant [40], and cholesterol-lowering [41] properties, which justify their potency.

Epidemiological studies suggest the existence of an inverse relationship between the high consumption of soybeans and of red clover isoflavones and cardiovascular risk [12,42,43]. In particular, several studies have shown that Asian females, whose consumption of soybeans and derived products is considered high, show a low frequency of post-menopausal symptoms (i.e., hot flushes) [44]. For this reason, isoflavones are currently being studied as estrogen-mimetics for hormone replacement therapy [45].

Several meta-analyses of the epidemiological and clinical trials conducted in Asian and Western populations published thus far have shown that the dietary intake of soy isoflavones lowers blood pressure sufficiently in hypertensive and pre-hypertensive subjects [46,47,48,49,50] to the point that they can lower overall cardiovascular risk [51]. Table 1 summarizes the main findings of the most recently published systematic reviews and meta-analyses on the anti-hypertensive effects of dietary intake of isoflavones.

Nonetheless, these epidemiological and clinical studies are limited by several factors, including (A) the chemical heterogeneity of the assessed foodstuffs/extracts/mixtures; and (B) the metabolism of isoflavones, which may itself depend on the age and gender of the subjects. The chemical composition of isoflavone-containing foodstuffs is known to depend on the botanical origin itself, but also on the food preparation process, as it was shown that isoflavone glycosides remain unmodified during several food preparation procedures [55,56]. For example, most soy-based foodstuffs have low levels of aglycones compared to glycosides, whereas fermented products show a higher aglycone content [57]. In addition, it is presently unclear whether isoflavones are handled similarly when consumed from different foodstuffs. It is thought that the interindividual differences in the proportion of intestinal bacteria may be responsible for a considerable fraction of such variability [58,59].

These factors increase the variability of the response, which hinders the understanding of what bioactive compounds may be responsible for the observed beneficial effects. Therefore, it becomes critical to assess the effect of the administration of isolated isoflavones on the cardiovascular system. This paper aims to review the main actions of isoflavones on the blood pressure-regulation mechanisms with particular focus given to the currently known mechanisms underlying their vasorelaxant effect. Several reviews, both comprehensive and systematic, have been published on the cardiovascular effects of isoflavones, including their vasorelaxant effects. However, to the author’s knowledge, the present review provides the most thorough mechanistic description of isoflavone-mediated vasorelaxation; it also covers more cellular pathways and includes more isoflavones than the previously published reviews. By providing such a thorough description of the currently known vascular mechanisms, as well as including several aspects on pharmacodynamics and pharmacokinetics, this review aims to establish a rationale for drug design as well as to improve the conception of future experimental studies.

## 2. Chemical Characterization, Metabolism, and Safety

**Figure 1:**
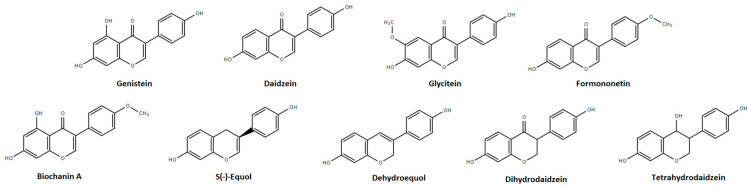
Molecular structure of the five main isoflavones in their aglycone form (genistein, daidzein, glycitein, formononetin, biochanin A) and of the metabolites S(−)-equol, dehydroequol, dihydrodaidzein, and tetrahydrodaidzein.

In general, isoflavone glycosides are not bioavailable and, thus, not biologically active. After ingestion, they are hydrolyzed into aglycones by the β-glucosidase enzymes present in the intestinal mucosa and in microbial flora, rendering isoflavones bioavailable [35,60]. Aglycones are then either absorbed or undergo further metabolism by intestinal flora or mucosa or by the liver before being excreted. The metabolic pathways for the majority of these isoflavones are well known in rats but less in humans. For all isoflavones, intestinal flora plays such an important role in their bioavailability that their effect has been shown to be highly dampened by an antibiotic treatment course [61]. The molecular structure of the isoflavones and biologically active metabolites discussed in this paper is shown in Figure 1.

Genistein (7,4′-dihydroxy-6-methoxyisoflavone), also known as genisteol or prunetol, has a molecular mass of 270.24 g/mol, an octanol-water partition coefficient (logP) of 3.04 [62] and low solubility in water (5.3 μM), the latter being responsible for its low bioavailability [63,64]. Ginestein is the aglycone form of genistin [65]. Genistein is absorbed in the gastrointestinal tract mainly by passive diffusion in a region-dependent manner, with higher absorption rates being found in the duodenum and colon and lower rates in the jejunum and terminal ileum [66]. Absorption may be limited by the breast cancer resistance protein (BCRP) [67,68], with the differential expression of this transporter in the gastrointestinal mucosa accounting, at least in part, for the observed heterogeneity in terms of absorption. For example, BCRP has a lower expression in the colon, which justifies the high absorption rate in that region [69]. The intestinal mucosa and the liver both contribute to the first-pass effect, metabolizing genistein to genistein-glucuronide and genistein-sulfate [70]. Genistein is finally excreted in the urine and feces as dihydrogenistein, 6′-OH-O-desmethylangolensin, trihydroxybenzene, and 3′,4′,5,7-tetrahydroxyisoflavone, as well as unmodified [70,71]. There are inconsistent results regarding its oral bioavailability in rats. It has been reported that genistein shows higher bioavailability than genistin in young non-anesthetized rats [65], which is in accordance to a previous study conducted in *caco-2* cells [72]. Another study, however, reported a higher bioavailability for genistein in rats of similar age and weight [64]. Nonetheless, oral administration of genistin shows comparable plasma levels compared to genistein administration alone, indicating rapid and complete intestinal hydrolysis, with no effect on absorption [69]. Gender is a factor that affects genistein absorption in rats, with females showing considerably higher rates than males [70,73]. In clinical studies, genistein shows moderate absorption after oral administration of soy supplements rich in genistein or genistin [74,75].

Daidzein (7,4′-dihydroxyisoflavone), also known as daidzeol, is a lipophilic compound with a molecular mass of 254.24 g/mol and a logP of 2.51 [62]. Daidzein is the aglycone form of both daidzin and puerarin [76]. In plants, daidzein is conjugated mainly with glucose, but also with 6″-O-malonyl or 6″-O-acetylglucose [77,78]. Daidzein is almost completely metabolized by the intestinal mucosa, intestinal flora, and by the liver [79]. In the intestine, bacteria metabolize daidzein to desmethylangolensin, dihydrodaidzein, and cis-4-OH-equol by demethylation and reduction processes [80]. Following this biotransformation, daidzein is converted in the liver into more hydrophilic products, such as glucuronide, sulfate, and sulfoglucuronide conjugates, which may affect its biological activity [81]. Daidzein is excreted unmodified in the urine [71] as well as in the form of other metabolites. The bioavailability of daidzein has been reported to be lower than that of genistein [82]. In the bloodstream, 80% of daidzein is transported bound to SHBG [83].

Glycitein (7,4′-dihydroxy-6-methoxyisoflavone) has a molecular mass of 284.26 g/mol, with a logP of 1.97 [62]. Glycitein undergoes little metabolism, mostly by intestinal bacteria that reduce it to dihydroglycitein, 2′,4′,4″-Trihydroxy-5′-methoxy-α-methyldeoxybenzoin (5′-OMe-O-dma), and 6-OMe-equol [71]. Glycitein is stable, because the immediate proximity of the 6-methoxyl and the 7-hydroxyl groups blocks the demethylation process. Therefore, glycitein is not converted to daidzein and does not generate equol [5].

Formononetin (7-hydroxy-4′-methoxyisoflavone) is a lipophilic compound with a molecular mass of 268.26 g/mol and a logP of 2.58 [84]. Due to its lipophilic character, it is absorbed via passive diffusion in the intestine, especially in the small intestine, showing a peak absorption at 30 min [85]. During first pass metabolism, formononetin is rapidly O-demethylated into daidzein before undergoing conjugation to glucuronides and/or sulfates [86]. Therefore, formononetin can also generate equol. In rats, formononetin was determined to have a half-life of ~2–3 h after oral administration and ~2 h after intravenous administration [85,87].

Biochanin A (5,7-dihydroxy-4′-methoxyisoflavone) has a molecular mass of 284.26 g/mol, and is deemed poorly water soluble (7 mg/mL) with no mention of a calculated logP in the literature [88]. Its poor oral bioavailability seems to be attributed to stomach degradation, susceptibility to hydrophilic degradation in the gastrointestinal tract, and extensive hepatic first-pass metabolism [86]. In rats, the metabolism of biochanin A is well known. In the intestine, bacteria demethylate biochanin A into genistein, which can then be metabolized in the liver to glucuronic and sulfonic conjugates, as well as undergo several hydroxylation reactions [37,89]. Biochanin A itself can undergo glucuronidation and sulfation, as well as hydroxylation. Finally, the hydroxylated products of biochanin A can themselves be demethylated into the hydroxylated products of genistein [89].

Equol (4′,7-dihydroxyisoflavan) is not an isoflavone but rather an isoflavan. It is a lipophilic metabolite of daidzein, with a molecular weight of 242.27 g/mol and a logP of 3.20 [62]. As a result of the chiral center at the C-3 carbon position, equol exists in two enantiomeric forms, *R*-(+)-equol and *S*-(−)-equol, with the latter being the natural diastereoisomer produced by intestinal bacteria in humans and rats [90]. In the bloodstream, 50% of equol is transported bound to SHBG [91]. Although not formally a phytoestrogen, it shares some structural features with estradiol, with its estrogenic properties having been suspected back in the 1940s, where the infertility observed in ewes grazing subterranean clover pastures in Australia, the so called “clover disease”, was attributed mainly to formononetin and biochanin A, with the former being converted into equol [92,93]. Although the majority of animals produce equol, only 20–50% human adults are capable of producing it in high amounts, as was observed in several studies following a soy challenge [35,80,94]. Thus, individuals have been classified as “equol producers” if their equol plasma concentration reaches values above 20 mg/L and as “equol non-producers” if their concentrations are lower than 10 mg/L [35]. The prevalence of equol producers varies according to geographical distribution, with an estimated 30–50% in Western populations and up to 60% in vegetarians or Asians [95], with high prevalence confirmed to depend on a high dietary intake [96]. These differences may depend on interindividual variability in terms of the composition of the intestinal flora as well on the composition of the diet itself [80]. Interestingly, it has been shown that short-term supplementation with isoflavones can stimulate the flora to produce equol and convert “equol non-producers” into “equol producers” [97]. Some authors have proposed that soy consumption only lowers cardiovascular risk in subjects that produce equol [35], whereas others have suggested that the cardiovascular benefit in equol-producers is not significant [98]. Still, it is known that the intestinal conversion of daidzein to equol is not necessary to exert its vasorelaxant activity in all vascular beds [99]. However, equol appears to have a slightly higher vascular antioxidant activity and a longer plasma half-life than daidzein in humans [100], which probably renders it more interesting as a therapeutic drug. Whole soy foods (less processed soy products, such as soy milk, soy nuts, soy flour, tofu, etc.) are more effective than isolated soy components and a purified single isoflavone is more effective than complex isoflavones [98].

Although generally perceived as safe, a considerable number of studies have highlighted several health risks associated with the intake of isoflavones, which must be taken into account. Prenatal and postnatal exposure to high levels of isoflavones has been associated with abnormalities in the reproductive organs in both genders, which may be irreversible and affect sexual function in adults [101,102,103]. Moreover, this exposure has been associated with an increased risk of uterine cancer [104] and with the probable risk of infant leukemias [105]. Prenatal exposure to isoflavones is possible since these compounds cross the placental barrier and access the fetal circulation [106]. Postnatal exposure is primarily due to consumption of soy-based infant formulae, soy milk, and soy food supplements [20,107], and not due to breast milk, which, irrespective of the dietary habits of the mother, excretes low levels of isoflavones [108]. Besides exposure, the apparent bioavailability of isoflavones in children is known to be higher than in adults, which contributes to these effects [109].

Several epidemiological studies have been published on the relation between the intake of soy-based products and the risk of dementia, with conflicting results. Although some studies have linked tofu and tempeh consumption to dementia in the elderly, the authors have attributed the presence of formaldehyde in one of the products to the increased risk of dementia, not isoflavones themselves [110]. Besides this, these studies also showed methodological limitations that preclude more consistent conclusions [111,112]. Several posterior clinical trials have shown more optimistic results but is still affected by a lack of internal consistency [113].

It has been established in vitro and in vivo that isoflavones, mainly genistein, can inhibit thyroid peroxidase, an enzyme involved in the biosynthesis of thyroid hormones [114]. In addition, genistein and, to a lower extent, daidzein compete with thyroxin in the attachment to transthyretin in vitro [115], the main transport protein for thyroid hormones. These effects might suppose that isoflavones change thyroid hormone homeostasis. However, given that an adequate iodine intake is present, there is no risk of thyroid disease, as found in children [116], healthy adults, pre- and post-menopausal women [117], and in hypothyroidism patients [118]. Still, studies describing the effects of isoflavones on subclinical hypothyroid patients and in iodine-deprived patients are lacking.

Several in vitro and preclinical studies have shown that isoflavones, mainly genistein, daidzein, and biochanin A, display interesting anti-cancer properties against several neoplasms, including prostate, pancreas, lung, skin, breast, and colon, among others [119]. Meta-analyses have indeed confirmed the protecting role of dietary isoflavones for prostate [120] and colorectal [121] cancers. However, there are conflicting results regarding other neoplasms, namely, breast and bladder cancers. Regarding bladder cancer, dietary intake of isoflavones has been both positively [122,123] and negatively correlated [124] with risk. Intake dose seems to be an important determinant of the histological progression and, therefore, of the prognosis of the disease [125]. Several studies have shown that dietary intake of isoflavones is associated with a lower risk of breast cancer [126,127,128]. In Asian countries, where the isoflavone intake is higher than in Western countries, the prevalence of breast cancer is lower [129,130]. It is also suggested that isoflavone intake during childhood or maturing may lower the risk of breast cancer in later years [131]. However, there are studies showing that consumption of soy foods has no protective effects against breast cancer [132]. Also, it appears that isoflavones may stimulate epithelial cell proliferation in the breasts of premenopausal women [133]. Regarding uterine cancer, the current results are inconclusive. Even though a high intake of anti-estrogenic isoflavones might constitute protection against cervical cancer [134]. One study reported lower isoflavone intake in a group of uterine cancer patients [135] while another found that a 6-month intake did not prevent cervical hyperplasia induced by exogenous estradiol [136]. Finally, in grazing animals, such as sheep and horses, the ingestion of large amounts of clover, rich in formononetin and biochanin A, causes “clover disease”, characterized by sexual dysfunction [92,137,138].

## 3. Vasorelaxant Effects of Isoflavones In Vitro

Vascular tone is defined as the basal degree of constriction sustained by each blood vessel when not subjected to any kind of stimuli. The increase in vascular tone (i.e., vasoconstriction) leads to an increase in vascular resistance, which leads to a downstream decrease in tissue perfusion and to an upstream increase in blood pressure. When vascular tone decreases (i.e., vasodilation), vascular resistance decreases, which leads to an increase in tissue perfusion and to a decrease in blood pressure [139,140]. Several mediators, neural, endocrine and local, contribute to the regulation of vascular tone. Neural mediators include neurotransmitters released by afferent nerve fibers, such as substance P and calcitonin gene-related peptide, as well as by the post-ganglionic sympathetic and parasympathetic nerve fibers, such as norepinephrine (NE)/epinephrine and acetylcholine (Ach), respectively. Endocrine mediators with important vasoactive effects include epinephrine/NE, angiotensin II, endothelin-1, vasopressin, insulin, among several others. Local mediators include endothelial substances, such as nitric oxide (NO); endothelium-derived hyperpolarization factors (EDHFs); autacoids, such as thromboxane A_2_ (TXA_2_); and mediators released by immune cells, including histamine, bradykinin, and substance P. Most of these mediators can exert either vasoconstriction or vasodilation, depending on the type of vessel and on the receptors they bind to [139,141], and change the vascular tone and caliber by acting either in the endothelium or in the vascular smooth muscle (VSM).

Several mediators, including NE/epinephrine, serotonin (5-HT), angiotensin II, vasopressin, and TXA_2_, act directly on the receptors localized on the VSM cell membrane which, via G proteins, open the receptor-operated calcium channels (ROCC). The entry of calcium from the extracellular fluid drives the release of more calcium from the endoplasmic reticulum. The resulting cytosolic calcium causes contraction by binding to calmodulin, which leads to the activation of myosin light chain kinase (MLCK), which changes the myofibrils spatial organization. Moreover, calcium influx directly causes VSM cell depolarization, which opens L-type-voltage-gated calcium channel (VGCC), further strengthening contraction [142]. Relaxation of the VSM cell occurs with the removal of calcium from the cytosol and with the activation of the myosin light chain phosphatase (MLCP), an enzyme that opposes the effects of MLCK on myofibrils. The activity of MLCP can be suppressed by the RhoA-associated kinase (ROCK) enzyme, favoring VSM contraction [143].

The endothelium constitutively expresses the endothelial isoform of nitric oxide synthase (eNOS), an enzyme that produces the powerful vasorelaxant NO. Due to its low molecular weight and to its lipophilic character, NO rapidly diffuses to VSM cells and activates the enzyme guanylyl cyclase, which, in turn, raises the intracellular cyclic guanosine monophosphate (cGMP) and leads to a change in myofibrils spatial organization, causing cell relaxation [144]. The levels and activity of eNOS can be affected by extracellular mediators (e.g., 17-β-estradiol) [145], by several intracellular mediators (e.g., calmodulin and caveolin-1) and pathways (e.g., PI3K/PTEN/Akt and MAPK) [144]. The endothelium also synthesizes mediators that cause hyperpolarization of the endothelial cells and/or of the VSM cells, probably by opening the potassium and/or chloride channels. The resulting potassium efflux and/or chloride influx cause hyperpolarization, which prevents the opening of VGCCs and of calcium influx. It is currently thought that those mediators, collectively termed EDHFs, include hydrogen peroxide, prostanoids, epoxyeicosatrienoic and eicosatrienoic acids (EETs and ETs, respectively) [146].

The vasorelaxant effects of a given test substance can be assessed by incubating it with a blood vessel ring in the presence of known substances that will either contract or relax that vessel via specific cellular pathways. Depending on whether it will potentiate or suppress the effects of other substances, the vascular mechanism of the test substance can then be inferred.

**Figure 2:**
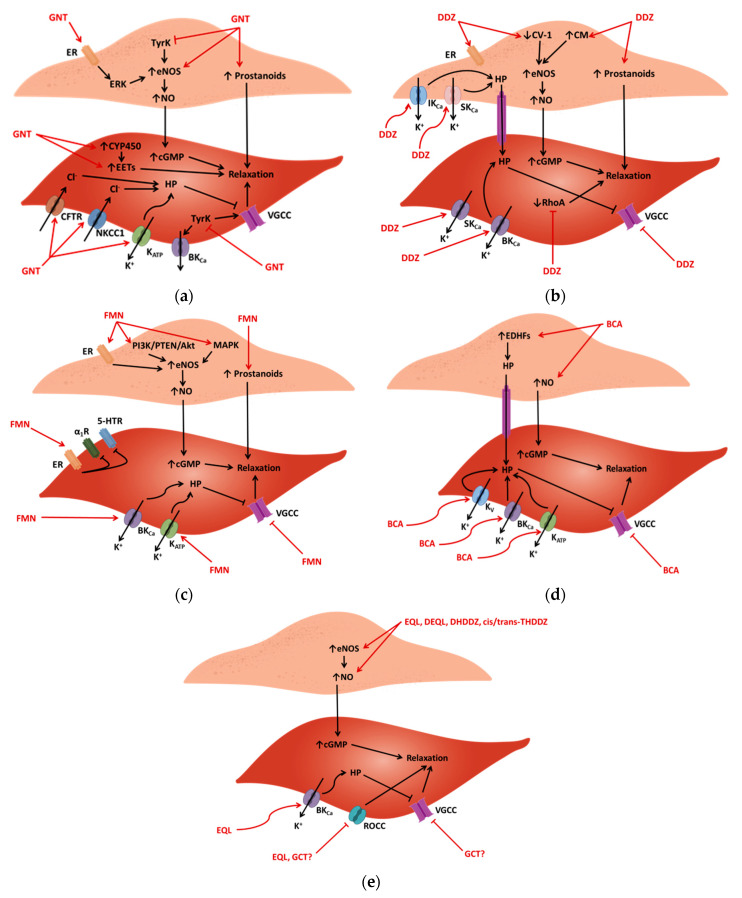
(**a**) Proposed vasorelaxation mechanisms for genistein (GNT); (**b**) proposed vasorelaxation mechanisms for daidzein (DDZ); (**c**) proposed vasorelaxation mechanisms for formononetin (FMN); (**d**) proposed vasorelaxation mechanisms for biochanin A (BCA); (**e**) proposed vasorelaxation mechanisms for glycitein (GCT), equol (EQL), dehydroequol (DEQL), dihydrodaidzein (DHDDZ), and cis- and trans-tetrahydrodaidzein (cis-/trans-THDDZ). 5-HTR—serotonin receptor; α_1_R—alpha-1 adrenergic receptor; cGMP—cyclic guanosine monophosphate; CFTR—cystic fibrosis transmembrane conductance regulator; CM—calmodulin; CV-1—caveolin-1; CYP450 – cytochrome P450 enzymes; EETs – epoxyeicosatrienoic acids; eNOS—endothelial nitric oxide synthase; EDHFs—endothelium-derived hyperpolarization factors; ER—estrogen receptor; HP—hyperpolarization; MAPK—mitogen associated protein kinase; NO—nitric oxide; ROCC—receptor-operated calcium channel; TyrK—tyrosine kinase; VGCC—voltage-gated calcium channel.

The vasorelaxant effects of isoflavones are attributed to multiple pathways, both in endothelial and in VSM cells and are described in Figure 2. There is a multitude of proposed mechanisms for genistein-induced vasorelaxation as this compound induces vasorelaxation in several vascular beds, including in rat aortae, carotid and pulmonary arteries preconstricted by several inducers, including phenylephrine (PE), potassium chloride (KCl), 5-HT, fluoride, phorbol ester, and TXA_2_ (Table 2) [79,147,148,149,150,151,152,153]. The proposed mechanisms for genistein-mediated vasorelaxation are shown in Figure 2a. Daidzein is known to relax several vascular beds, including the aortic, mesenteric and basilar arteries by both endothelium-dependent and endothelium-independent mechanisms, which do not seem to be site-specific (Table 3). The proposed mechanisms for daidzein-mediated vasorelaxation are shown in Figure 2b. Formononetin is known to relax several vascular beds, including the aorta, mesenteric, renal, coronary, and cerebral arteries [154]; however, most studies have been performed in the first two, where the relaxation response is more potent, having led to a deeper comprehension of endothelium-dependent and –independent mechanisms (Table 4). The proposed mechanisms for formononetin-mediated vasorelaxation are shown in Figure 2c. Two studies have reported that biochanin A is the most potent vasorelaxant of the major phytoestrogen isoflavones [38,155]. It induces vasorelaxation by acting both in the endothelium and in VSM cells. Most studies have been conducted in the aortae of healthy and diseased animals. In healthy animals, results suggest that biochanin A-mediated vasodilation is endothelium-independent, whereas in diseased animals it seems to depend on the pathophysiology of the disease itself (Table 5). The proposed mechanisms for biochanin A-mediated vasorelaxation are shown in Figure 2d. Few studies on the vasorelaxation evoked by glycitein or by the metabolites equol, deydroequol, dihydrodaidzein and tetrahydrodaidzein are available (Table 6). Current knowledge suggests that these compounds act in the endothelium and in VSM cells, and their proposed mechanisms of vasorelaxation are shown in Figure 2e.

### 3.1. Endothelium-Dependent Vasorelaxation 

In healthy Wistar rats, vasorelaxation of the aorta and pulmonary artery preconstricted by PE and KCl is attenuated by endothelium denudation and by L-N^ω^-nitro arginine methyl ester (L-NAME, i.e., NOS inhibitor) but not by indomethacin (i.e., cyclooxygenase, COX, inhibitor) [149], showing that in this strain and vessels the endothelial prostanoid pathway is not relevant. In female ovariectomized (i.e., low estrogen levels) Sprague-Dawley rats, genistein restores endothelial-dependent vasorelaxation, which is attributed to a direct increase in eNOS activity [156]. The potentiation of the NO/cGMP pathway is also of relevance for the relaxation of human umbilical veins preconstricted with 5-HT, since L-NAME partially attenuates the response [157].

In healthy Wistar–Kyoto rats, vasorelaxation of PE- and KCl-preconstricted arteries by daidzein is reduced by endothelium denudation, by L-NAME, or indomethacin, showing the importance of endothelial NO/cGMP and prostanoid pathways for this response [150]. In the aortae of Sprague-Dawley and of Wistar rats, daidzein evokes an endothelium-dependent vasorelaxation, both in healthy and in diseased animals. In healthy Wistar rats, daidzein relaxes PE-preconstricted aortae, an effect that is attenuated by L-NAME, by endothelial denudation, but not by ICI 182,780 (fulvestrant, i.e., estrogen receptor antagonist) [149]. In diabetic Wistar rats, daidzein also relaxes PE-preconstricted aortae and potentiates Ach-induced vasorelaxation, an effect that was decreased by L-NAME and indomethacin [158]. In L-NAME hypertensive Wistar rats, daidzein relaxes KCl-preconstricted aortae and potentiates Ach-induced relaxation [159]. These studies suggest that daidzein stimulates the endothelial NO/cGMP and prostanoid pathways directly. In the aortae of healthy Sprague-Dawley rats, daidzein again potentiates Ach-induced vasorelaxation [160,161,162] but does not affect sodium nitroprusside- (SNP, i.e., NO donor) or isoproterenol (i.e., beta adrenergic receptor agonist)-induced vasorelaxation [160,162], suggesting that it increases both basal and stimulated NO secretion. Here, the explanation for this increase in NO is not the modulation of eNOS activity. Rather, it is the decrease in the expression of caveolin-1 and the increase in the expression of calmodulin that accounts for the increased expression of eNOS [160,161,162]. In endothelial cells, caveolin-1 binds eNOS and restricts its activity [163], while calmodulin prevents that binding [164]. Therefore, besides stimulating the NO/cGMP pathway directly, daidzein also increases the endothelial availability of eNOS by inhibiting caveolin-1. This modulating effect of caveolin-1 might be attributed to the activation of the estrogen receptor, since estrogen itself upregulates eNOS [165] and downregulates caveolin-1 [166], whereas ovariectomy upregulates caveolin-1 [162]. However, this modulating effect of estrogen in daidzein-mediated vasorelaxation is not verified in all strains, let alone in all vascular beds. For example, in female ovariectomized Sprague-Dawley rats, daidzein prevents the decrease in the endothelial function associated with the decline in the estrogen levels [162].

In Wistar rats, formononetin relaxes PE- and 9,11-Dideoxy-9a, 11a-methanoepoxy prostaglandin F2α (U46619, i.e., thromboxane TXA_2_ mimetic)-preconstricted intact aortae, a response which is suppressed by L-NAME, methylene blue (i.e., guanylyl cyclase inhibitor), and endothelium denudation, but not by N-(3-(Aminomethyl)benzyl)acetamidine (1400 W, i.e., inhibitor of the inducible isoform of nitric oxide synthase, iNOS) or propranolol (i.e., nonselective beta adrenergic receptor blocker) [154]. In denuded aortae, methylene blue reduces but does not abolish vasorelaxation, showing that VSM guanylyl cyclase is essential for relaxation and that there are also endothelium-independent mechanisms as well [167]. In Wistar rat mesenteric arteries precontracted by PE and U46619, formononetin-mediated vasorelaxation is partially attenuated by L-NAME and endothelium denudation, whereas indomethacin shows little inhibiting effects. Taken collectively, these results suggest that the endothelial NO/cGMP pathway is important for formononetin-mediated vasorelaxation, although it seems to be site-specific, with the endothelial prostanoids showing relevance in the mesenteric arteries but not in the aorta [154].

In addition to a direct stimulation, formononetin may potentiate the endothelial NO/cGMP pathway by upregulating eNOS at both the mRNA and protein levels [167]. In human umbilical vein endothelial cells (HUVECs), formononetin increases NO production and upregulates the expression of eNOS and of neuronal NOS (nNOS) without affecting iNOS [168]. Formononetin is able to increase eNOS expression via activation of the PI3K/PTEN/Akt and MAPK pathways. The enzyme PI3K catalyzes the production of phosphatidylinositol-1,4,5-triphosphate (PIP_3_) from phosphatidylinositol-4,5-biphosphate (PIP_2_), resulting in the phosphorylation of Akt and other downstream substrates. Phosphatase and tensin homolog deleted on chromosome 10 (PTEN) is a regulatory enzyme that dephosphorylates PIP_3_, leading to Akt suppression. Formononetin binds PTEN, preventing the suppression of the PI3K/PTEN/Atk pathway, therefore contributing to vasorelaxation [169]. In the rat mesenteric arteries, formononetin-mediated relaxation is partly attributed to the upregulation of eNOS, since it is attenuated by ICI 182,780 [170]. In this vascular bed, formononetin upregulates eNOS through MAPK pathways. The MAPK family is composed of three proteins, ERK, JNK, and p38. ERK alone is unable to upregulate eNOS [171], whereas the combined activation of ERK and JNK do enhance eNOS expression. Protein p38 acts in the opposite direction, suppressing eNOS upregulation [172,173]. Formononetin enhances eNOS expression through activation of the estrogen receptor and both the ERK and JNK downstream proteins [170]. In fact, estrogen can improve vascular endothelial function through upregulation of eNOS and release of NO [174].

In SHRs, biochanin A-induced vasorelaxation is endothelium-dependent, being attenuated by endothelial denudation but not by L-NAME or indomethacin. This suggests the involvement of an EDHF other than NO and prostanoids [175]. In the aortae of two-kidney–one-clip (2K1C) renovascular hypertensive rats, biochanin A again appears to induce endothelial-dependent vasorelaxation since endothelium removal decreases the potency of the response. Since biochanin A neither potentiates Ach or SNP-induced vasorelaxation nor is affected by L-NAME, it is probable that vasorelaxation is also mediated by EDHFs [176]. In porcine coronary arteries, biochanin A potentiates vasorelaxation evoked by SNP but not by forskolin (i.e., ROCCs blocker) which suggests that in this vascular bed it raised the intracellular concentration of cGMP [177].

The vasoactive effects of equol, dehydroequol, dihydrodaidzein, cis-tetrahydrodiadzein and trans-tetrahydrodiadzein were examined in the aortae of Sprague-Dawley rats. They showed endothelium-dependent vasodilation, inhibited by endothelium denudation, N^ω^-NO-nitro-L-arginine (NOLA, i.e., NOS inhibitor), 1H-[1,2,4]oxadiazolo [4,3-a]quinoxalin-1-one (ODQ, i.e., selective inhibitor of NO-sensitive guanylyl cyclase), and KCl. Contrarily to its effects in most vascular beds, 17β-estradiol showed endothelium-independent vasodilation [178]. Equol evokes an endothelium-dependent vasorelaxation by stimulating NO release, which is not affected by the ER antagonist ICI 182,780 nor by pertussis toxin (i.e., protein Gi inhibitor) [179]. In a model of endothelial dysfunction, ovariectomized rats were deprived of dietary intake of isoflavones. In these animals, the Ach- and A23187 (i.e., calcium ionophore)-induced endothelium-dependent vasorelaxation were compromised and the administration of equol partially restored that response. Equol did not affect SNP-induced vasorelaxation. Moreover, an ovariectomy decreased the expression of eNOS, which equol partially restored. These results show that equol has predominantly endothelium-dependent vasorelaxant effects, consisting of the potentiation of NO synthesis [180]. In human uterine arteries, equol attenuates PE- but not KCl-induced contraction, suggesting that it blocks ROCCs but not VGCCs [181]. Equol also reverses the endothelial dysfunction caused by ritonavir (i.e., HIV protease inhibitor) in porcine pulmonary arteries. Ritonavir reduces eNOS expression, reduces bradykinin-induced vasorelaxation as well as U46619-induced contraction, whereas equol normalized all these responses [182].

### 3.2. Endothelium-Independent Vasorelaxation—The Role of Calcium Channels

Endothelium-independent vasorelaxation mechanisms have been identified for genistein in several vascular beds. In rat mesenteric arteries, genistein relaxes NE-, KCl-, and CaCl_2_ preconstricted vessels, responses which are not affected by L-NAME or indomethacin, ruling out the importance of the endothelial NO/cGMP and prostanoid pathways. In this case, genistein seems to act directly in VSM cells. Considering that NE leads to an increase in cyclic adenosine monophosphate (cAMP) levels and that KCl and CaCl_2_ both lead to VSM cell depolarization and VGCC activation, it has been proposed that genistein-mediated vasorelaxation involves the protein kinase A (PKA) pathway [183] or the activation of VGCCs [148].

In the basilar artery of New Zealand rabbits, daidzein relaxes KCl- and uridine 5’-triphosphate (UTP)-preconstricted vessels, a response that is not affected by L-NAME, ODQ, 4H-8-bromo-1,2,4-oxadiazolo(3,4-d)benz (b)(1,4)oxazin-1-one (NS2028, i.e., specific soluble guanylyl cyclase), or indomethacin. This suggests that no endothelial mediators, NO, or EDHFs are involved in this response. Given that daidzein relaxes the CaCl_2_-preconstricted arteries, it seems to act as a VGCC blocker in VSM cells [155].

Little is known of the vasorelaxation activity of isolated glycitein, given that it has been mainly studied as a part of mixed isoflavone preparations. It is known to relax NE-preconstricted aortae from Wistar Han rats, suggesting it interferes with the calcium influx. In porcine coronary arteries preconstricted by 9,11-Dideoxy-9a, 11a-methanoepoxy prostaglandin F2α (U46619, i.e., thromboxane TXA_2_ mimetic), glycitein evokes vasorelaxation in an endothelium-independent way. Furthermore, it attenuates KCl-, calcium chloride (CaCl_2_)-, 5-HT-, and U46619-induced constriction of coronary arteries in only a partial way and at high concentrations [177]. More studies are necessary to clarify the vasorelaxation mechanism of glycitein.

Again, in Wistar rats, formononetin relaxes KCl- and NE-preconstricted aortae, by preventing extracellular calcium influx and by inhibiting the calcium release intracellular stores, respectively. Thus, formononetin also acts on VGCCs and ROCCs on the VSM plasma membrane [184]. Formononetin relaxes KCl- and CaCl_2_-preconstricted Wistar mesenteric arteries, again suggesting that it may inhibit VGCCs. Furthermore, because FMN inhibits PE-induced contraction in a calcium-free medium, it inhibits calcium release from intracellular stores in VSM cells, but does not inhibit the extracellular calcium influx through receptor-operated calcium channels. Finally, formononetin does not relax caffeine-contracted vessels, implying that ryanodine receptors are not involved in this intracellular calcium release, but rather inositol 1,4,5-trisphosphate (IP_3_) receptors [154].

In healthy Sprague-Dawley rats, biochanin A relaxes PE-, KCl-, and CaCl_2_-preconstricted aortae, both intact and denuded alike, suggesting that the effect is endothelium-independent, involving VSM cells directly [185]. Similarly, in healthy Wistar Han rats, biochanin A relaxes NE-preconstricted aortae, both intact and denuded alike [177]. Since it inhibits NE-, PE-, and KCl-induced contraction, biochanin A may inhibit the influx of extracellular calcium into VSM cells from ROCCs or VGCCs, and/or inhibit the release of calcium from intracellular stores [185]. In endothelium-denuded porcine coronary arteries, biochanin A blocks the effects of Bay K8644 (i.e., L-type calcium channel activator), showing that it blocks the VGCCs on VSM cells [177].

Again, in Sprague-Dawley rats, equol relaxes the carotid artery in an endothelium-independent manner, given that L-NAME and endothelium denudation did not affect the response. Equol-mediated vasorelaxation was also unaffected by incubation with U46619 or KCl with nimodipine (i.e., L-type calcium channel blocker), showing that calcium and potassium channels are not involved in this vascular bed [99]. However, in PE-preconstricted rat aortae, equol-mediated relaxation suggests that it may interfere with the receptor-mediated calcium channels [186].

### 3.3. Endothelium-Independent Vasorelaxation—The Role of Potassium Channels

Different types of potassium ion channels also seem to be involved in genistein-mediated vasorelaxation. In rabbit pulmonary artery cells, genistein is known to block voltage-gated potassium channels [187]. In human umbilical veins preconstricted with 5-HT, genistein-mediated vasorelaxation is decreased by glibenclamide (i.e., K_ATP_ channel blocker), suggesting that genistein opens the K_ATP_ channels in the VSM cell membrane, leading to hyperpolarization and relaxation [157].

The modulation of endothelial and VSM potassium channels are also implicated in endothelial-independent vasorelaxation of the rat aortae and mesenteric arteries. In Wistar rat aortae, daidzein (7 days) potentiates Ach-mediated vasorelaxation, an effect that is attenuated by L-NOLA alone or in combination with hemoglobin. This vasorelaxation is also attenuated by PPOH (i.e., selective inhibitor of the cytochrome P450 isoenzymes epoxygenation reactions) but not by indomethacin. This indicates the existence of endothelium-dependent relaxation mechanisms, which involve both NO/cGMP as well as EDHFs, and probably EETs, products of epoxygenase enzymes that facilitate the opening of the endothelial potassium channels. In fact, daidzein seems to open several potassium channels on VSM and endothelial cells, since charybdotoxin (i.e., large-conductance and intermediate-conductance voltage- and calcium-activated potassium channels, BK_Ca_ and IK_Ca_, blocker), apamin (i.e., small-conductance calcium-activated potassium channels, SK_Ca_, blocker), barium (i.e., inward-rectifier potassium channels, K_IR_, blocker), and oubain (i.e., sodium/potassium-ATPase blocker) also attenuate the potentiation of Ach-induced relaxation. In short, daidzein may open the SK_Ca_ and IK_Ca_ channels on the endothelial cells, probably reinforced by the increased synthesis of EETs. The increased efflux of potassium by these channels can open the K_IR_ channels and sodium/potassium-ATPase in VSM cells, leading to their hyperpolarization. The opening of BK_Ca_ channels on a VSM membrane can further accentuate this hyperpolarization [188]. In Wistar rat mesenteric arteries, daidzein induces vasorelaxation of NE-, KCl-, or CaCl_2_-precontracted mesenteric arteries, suggesting an action that prevents calcium entry, either through receptor-operated or voltage-gated channels. This response is not affected by removal of endothelium nor by indomethacin or L-NAME, besides being also gender-independent, which rules out the influence of NO/cGMP, prostanoids, and estradiol in modulating the tone of this vascular bed [148]. Posterior studies have highlighted a role for potassium channels, in particular BK_Ca_ channels, with the added knowledge that the beta-1 subunit of these channels is important for daidzein vasorelaxation, but only required if the substance is applied from the extracellular side of the membrane [189]. Furthermore, relaxation of NE- and KCl-preconstricted mesenteric arteries by daidzein is inhibited by iberiotoxin (i.e., a BK_Ca_ channel blocker), charybdotoxin, and by apamin, whereas 4-aminopyridine (i.e., blocker of voltage-gated K^+^ channels, K_V_), glibenclamide, or barium have no effect on this vasorelaxation. This study revealed that, in mesenteric arteries, daidzein opens the BK_Ca_ and SK_Ca_ channels in VSM cells, inducing hyperpolarization and relaxation [190]. Finally, one other study also mentioned the relaxation of daidzein in the carotid as well as basilar arteries, but did not explore the underlying mechanisms of relaxation [99].

In Wistar rats, propranolol, ICI 182,780 and mifepristone (i.e., progesterone receptor antagonist) fail to antagonize formononetin-induced aortic vasorelaxation, meaning that the compound does not appear to block the beta adrenergic, estrogen, or progesterone receptors on VSM cells. In contrast, glibenclamide and iberiotoxin suppress formononetin-mediated vasorelaxation in denuded vessels, showing that the opening of potassium channels in VSM cells constitutes a mechanism of endothelial-independent vasorelaxation [167]. In rat mesenteric arteries, the BK_Ca_ channel activation appears to be more prominent in intact vessels rather than in denuded ones, suggesting that it has an important role in the endothelium, besides increasing NO secretion [168]. This directly contrasts with the role of the BK_Ca_ channels on the rat aorta, whose activation was found only in denuded vessels (VSM cells). These discrepancies may be attributed to anatomical variations in the vasoreactivity to drugs [191]. In particular, endothelium-independent vasoactivity is more pronounced in the smaller resistance arteries, such as the mesenteric arteries, than in large conduits, such as the aorta [192,193], where endothelium-dependent activities prevail.

In healthy Sprague-Dawley rats, biochanin A-mediated vasorelaxation is inhibited by tetraethylammonium (TEA, i.e., BK_Ca_ channel blocker) and glibenclamide but not by 4-aminopyridine, suggesting that biochanin A-induced vasorelaxation involves the activation of the BK_Ca_ and K_ATP_ channels in VSM cells, with consequent hyperpolarization [185]. Similar results were found in the aortae of healthy Wistar–Kyoto rats, where biochanin A induced endothelium-independent vasorelaxation by opening the K_ATP_ or voltage-gated potassium channels [175]. Similarly, in SHRs, glibenclamide and 4-aminopyridine also attenuated the vasorelaxation response [175]. In the aortae of healthy Sprague-Dawley rats undergoing a sham surgery to the renal artery, biochanin A appears to induce endothelial-independent vasorelaxation since endothelium denudation does not affect the potency of the response. Glibenclamide, TEA, and 4-aminopyridine inhibit vasorelaxation, suggesting that opening of the BK_Ca_, K_ATP_, and K_V_ channels mediate the endothelium-independent mechanism [176]. In the aorta of 2K1C rats endothelium denudation significantly reduces biochanin A-mediated vasorelaxation. Glibenclamide, TEA and 4-aminopyridine also inhibit vasorelaxation, once again showing the involvement of BK_Ca_, K_ATP_, and K_V_ channels [176]. In the basilar artery of healthy rabbits, biochanin A-mediated vasorelaxation is endothelium-independent, given that the response is similar in intact and in denuded vessels. Because L-NAME, indomethacin, ODQ, and NS2028 do not affect vasorelaxation, it does not seem to involve NO or prostanoids [155]. In the rabbit coronary arteries, biochanin A mediated a concentration-dependent vasorelaxation, which is both sex- and endothelium-independent, although a mechanism has not been proposed by the authors [38].

In the high insulin-treated carotid artery of male Wistar rats, equol evokes vasorelaxation, a response blocked by iberiotoxin. Moreover, equol attenuated the contraction evoked by TCB2 (i.e., a selective 5-HT_2A_ receptor agonist) but not by BW723C86 (i.e., selective 5-HT_2B_ receptor agonist). These results suggest that, in this vascular bed, equol prevents 5-HT-mediated vasoconstriction via BK_Ca_ channel blockade [194]. In rat basilar arteries, equol evokes vasorelaxation, which is suppressed by paxilline (i.e., selective BK_Ca_ channel blocker) and iberiotoxin. This suggests that equol relaxes VSM cells by evoking hyperpolarization [195].

### 3.4. Endothelium-Independent Vasorelaxation—The Role of Chloride Channels

Direct VSM cell hyperpolarization via anion influx is a mechanism of genistein-mediated vasorelaxation present in the rat and mouse aortae. Genistein activates the cystic fibrosis transmembrane conductance regulator (CFTR) chloride channel and increases the flux of anions across the membrane [196]. Moreover, in denuded rat aortae, the vasorelaxant effect of genistein is inhibited by diphenylamine-2-carboxylic acid (i.e., CFTR inhibitor), glibenclamide, H-89 (i.e., selective PKA inhibitor), and bumetanide (i.e., NKCC1 inhibitor). These results strongly suggest that, in rat aortic VSM cells, the genistein-mediated vasorelaxation requires cAMP-dependent phosphorylation of CFTR and the entry of chloride ions via the NKCC1 cotransporter [197].

### 3.5. Potentiation of the Protein Kinase A Pathway

In male Wistar rats, genistein potentiates the relaxation effect of isoproterenol, forskolin, and dibutyryl cAMP in PE-preconstricted aortae. The potentiation of forskolin-induced vasorelaxation is inhibited by theophylline (i.e., phosphodiesterase inhibitor), quinacrine (i.e., phospholipase A_2_ inhibitor), and iberiotoxin. These results suggest that genistein-induced aortic vasorelaxation also occurs via potentiation of phospholipase A_2_ and PKA pathways. Moreover, the potentiation of isoproterenol vasorelaxation is inhibited by α-naphthoflavone and 8-methoxypsoralen (i.e., type I inhibitors of cytochrome P-450), suggesting that EEAs are mediators of this vasorelaxation response [198].

### 3.6. Role of Tyrosine Kinase Inhibition

Several studies have shown the involvement of tyrosine kinase activation in vascular constriction to several inducers and, conversely, the role of tyrosine kinase inhibition in the vasorelaxation response. Tyrosine kinase is known to modulate the action of different types of cation channels, in particular calcium channels. For example, tyrosine kinase modulates the activation of receptor-operated calcium channels in NE-mediated vasoconstriction [199], the calcium influx across nonselective cation channels associated with muscarinic receptors [200], and the conformation of VGCCs in VSM cells, keeping them in an available state for activation by depolarization [201]. This enzyme may also be involved in modulating the generation of endothelial NO [202]. Among all isoflavone phytoestrogens, genistein is the only one currently believed to act as a tyrosine kinase inhibitor [38,160,203]. In the aorta of spontaneously hypertensive rats (SHRs), tyrosine kinase may play a regulatory role in smooth muscle contraction and endothelium-dependent relaxation [204]. It is thought that in ovariectomized hypertensive rats genistein attenuates constriction of the renal artery by inhibiting tyrosine kinase [205]. In the basilar artery, genistein and tyrphostin 47 (i.e., tyrosine kinase inhibitor) alike attenuate the vasorelaxation to Ach and bradykinin but not to SNP. This suggests that the genistein-mediated vasorelaxation of the basilar artery involves inhibition of tyrosine kinase, which therefore leads to activation of the NO/cGMP pathway [202].

In porcine coronary arteries, genistein enhances the endothelium-independent action of SNP and levcromakalim (i.e., K_ATP_ channel activator), but not that of the endothelium-dependent bradikynin [206]. When denuded porcine coronary arteries are dilated by levcromakalin, genistein is able to restore this response in the presence of mexiletine but not lidocaine (i.e., voltage-gated sodium channel blocker). This suggests that the tyrosine kinase inhibiting activity of genistein was partially involved in the restoration of that vasorelaxation via the K_ATP_ channels [207].

Genistein is also able to correct vascular hyporesponsiveness, hypotension, and endothelial dysfunction in different contexts. For example, it can correct the vascular hyporeactivity of mesenteric arteries following a hemorrhagic shock. This hyporeactivity is associated with the phosphorylation of the alpha subunit of the BK_Ca_ channels, which probably facilitates their opening and, therefore, increases vasorelaxation. By inhibiting tyrosine kinase-mediated phosphorylation of BK_Ca_, genistein probably keeps these channels closed, increasing vascular reactivity to contractile stimuli [208]. Administration of genistein suppresses lipopolysaccharide (LPS)-induced long-term hypotension, endothelial dysfunction, and vascular hyporesponsiveness to NE in conscious rats. The main mechanisms underlying this effect are the inhibition of iNOS and a reduction in the tissues’ oxidative status [209,210,211]. Since tyrosine phosphorylation is a step in the LPS pathway, its inhibition by genistein appears to prevent iNOS induction in VSM cells and improve vascular reactivity [209].

Another vascular mechanism of genistein to consider deals with angiotensin II. The signal transduction pathway of angiotensin II leads to the activation of tyrosine kinase [212]. Since genistein itself can inhibit the expression of angiotensin converting enzyme (ACE) [213], it is quite possible that some of its beneficial effects on the vasculature result from the inhibition of both molecular targets.

In several studies in pulmonary arteries and veins, genistein induced vasorelaxation to the contraction induced by several agonists via tyrosine kinase inhibition. It is thought that one of the targets of this tyrosine kinase phosphorylation are regulatory proteins of the Rho kinase enzyme, which modulates the sensitivity of VSM cells to calcium [214]. Finally, recent studies in pulmonary vessels have shown that genistein ameliorates pulmonary hypertension via downregulation of the estrogen receptors [215,216].

### 3.7. Inhibition of Rho-Kinase

In male Sprague-Dawley rats, genistein completely relaxes aortae preconstricted with both full (fluoride) or partial (KCl, phorbol ester, PE or TXA_2_) RhoA/Rho-kinase activators. When Y-27632 (i.e., Rho-kinase inhibitor) is co-administrated with genistein to fluoride-preconstricted aortae, it fails to potentiate the relaxation effect of the genistein itself. Furthermore, genistein decreases phosphorylation of myosin phosphatase target subunit 1 (MYPT1) at Thr855 induced by U46619. Taken together, these results lead to the hypothesis that genistein decreases the Rho-kinase activity, thereby leading to vasorelaxation. Rho-kinase phosphorylates MLCP, decreasing its activity and leading to the buildup of phosphorylated myosin light chains. In addition, Rho-kinase can also phosphorylate myosin light chains directly and independently of the kinase and phosphatase activities. Relaxation of denuded vessels was not inhibited by iberiotoxin, thus ruling out the involvement of BK_Ca_ channels [152]. In another study, genistein prevented the lysophosphatidylcholine-induced contraction of rat aortae by inhibition of tyrosine kinase and consequent prevention of an increase in intracellular calcium [217]. In SHRs, genistein potentiates Y-27632 (i.e., Rho kinase inhibitor)-mediated vasorelaxation, which would suggest that an inhibition of Rho kinase was important for this response. However, genistein did not attenuate the increased Rho kinase activity, suggesting that it did not act directly upon this enzyme but probably on tyrosine kinase itself [218]. It is known that the Rho-kinase and tyrosine kinase pathways are intertwined in terms of regulation of vascular tone, although it is presently unclear which pathway exists upstream of the other [152].

In Sprague-Dawley rats, daidzein partially attenuates the aortic contraction to several inductors of RhoA kinase, including PE-, phorbol ester-, KCl-, and TXA_2_-preconstricted aortas, while exerting full relaxation to fluoride-preconstricted vessels. This marks a difference in its vasorelaxation mechanism with regard to genistein, which shows no difference in the magnitude of relaxation to these inductors. In addition to blocking calcium channels, these results also suggest that daidzein may inhibit RhoA kinase, or phosphorylate of extracellular signal-regulated kinase, a protein kinase C-potentiated inhibitory protein for protein phosphatase type 1 or integrin-linked kinase [152].

### 3.8. Activity on Estrogen and Epidermal Growth Factor Receptors

Among the studied isoflavones, genistein binds to the estrogen receptor with the highest affinity, preferentially to the β subtype [219,220], while daidzein and glycitein show lower but comparative binding affinities [221]. Dadizein benefits from a low-affinity binding to estrogen receptor α/β subtypes [160]. Vascular estrogen receptors are located in both endothelial and VSM cells [222]. Apparently, the activation of membrane estrogen receptor in VSM cells is linked to the activation of epidermal growth factor receptor (EGFR), which constitutes yet another mechanism for vasorelaxation by genistein. In the aortae of SHRs, genistein potentiates the Ach- and A23187-induced vasorelaxation. This response is not inhibited by ICI 182,780, but is inhibited by MPP (i.e., specific estrogen receptor subtype α antagonist) and by AG1478 (i.e., EGFR inhibitor). Thus, genistein apparently interacts with estrogen receptor subtype α and, via the respective G protein pathway, transactivates EGFR and leads to activation of extracellular signal-regulated kinase. This will ultimately increase eNOS phosphorylation and, consequently, lead to the increase in NO release [223,224].

### 3.9. Effect of Adrenergic Receptors

The chronic administration of formononetin seems to relax the mesenteric arteries in male SHRs by a different mechanism. After the oral administration of formononetin (50 mg/kg/day) for 8 weeks, the constriction of mesenteric arteries to PE- and 5-HT, whose receptors are overexpressed in hypertension, is attenuated. This response is associated with a decrease in the expression of the alpha-1 adrenoceptors and 5-HT_2A/1B_ receptors at both the mRNA and protein levels, with the accompanying increase in NE and 5-HT levels in plasma. Moreover, the eNOS expression was increased. In addition, although also increased in hypertension, endothelin-1 and U46619 do not seem to participate in this relaxation, since formononetin fails to relax the arteries when exposed to these substances. Taken together, these results suggest that the chronic administration of formononetin relaxes the mesenteric arteries of SHRs, apparently due to the combined downregulation of the alpha-1 adrenoceptors and 5-HT_2A/1B_ receptors and upregulation of eNOS [225]. The downregulation of the alpha-1 adrenergic receptors is theorized to be attributed to the estrogen-like property of formononetin, since estrogen itself can downregulate the alpha-1 receptors in urethral smooth muscle in rats [226] and formononetin is more potent than estrogen itself at modulating gene expression [227]. 

**Table 2:** Description and main results of in vitro studies on the vasorelaxant activity of genistein.

Authors	Compound Concentration	Species and Strain	Type of Vessel	Main Results
Laniyonu et al. (1994) [228]	1–15 μM	Male Sprague-Dawley rats	Aorta	Relaxation of pervanadate-induced but not of KCl-induced contraction
Moritoki et al. (1995) [211]	100 μM	Rat (undisclosed strain)	Aorta	Prevention of LPS-primed, L-arginine-mediated vasorelaxation
Filipeanu et al. (1995) [153]	10^−6^–10^−3.5^ M	Male Wistar rats	Aorta	Relaxation of PE-preconstricted vessels
Herrera et al. (1996) [229]	10^−6^–10^−3^ M	Wistar rats (both genders)	Aorta	Relaxation of NE-, KCl-, phorbol 12-myristate-13-acetate-preconstricted vessels, responses found to be independent of tyrosine kinase inhibition
Watts et al. (1996) [147]	5 × 10^−6^ M	Male Sprague-Dawley rats	Carotid artery and aorta (denuded)	Relaxation of 5-HT-preconstricted denuded carotid artery but absence of relaxation of phorbol-12,13-dibutyrate or KCl-preconstricted vessels
Duarte et al. (1997) [209]	Genistein (10 mg/kg i.p.)	Male Wistar rats	Aorta	Inhibition of LPS-mediated hyporresponsiveness to NE and inhibition of nitrite accumulation without affecting NOS.
Satake et al. (1999) [198]	3 × 10^−7^, 10^−6^, 10^−5^ M	Male Wistar rats	Aorta	Relaxation of PE-preconstricted vessels. Potentiation of isoproterenol, forskolin and dibutyryl cAMP-induced vasorelaxation Isoproterenol-mediated vasorelaxation was inhibited by α-naphthoflavone and by 8-methoxypsoralen; the potentiation of forskolin-mediated vasorelaxation was inhibited by theophylline, iberiotoxin and quinacrine
Squadrito et al. (2000) [156]	0.2 mg/kg/day administered subcutaneously for 4 weeks	Ovariectomized mature female Sprague-Dawley rats	Aorta	Ovariectomy decreased Ach-induced vasorelaxation but did not affect SNP response. Genistein restored endothelial-dependent vasorelaxation as well as N^ω^-L-arginine (L-NMA)-induced contraction.
Mishra et al. (2000) [149]	1–100 μM	Male Wistar rats	Aorta and pulmonary arteries	Relaxation of PE and KCl-preconstricted vessels, attenuated by endothelium denudation and L-NAME but not by indomethacin.
Suenaga et al. (2002) [217]	3 × 10^−6^, 10^−5^, 3 × 10^−5^ M	Male Wistar rats	Aorta	Prevention of lysophosphatidylcholine-induced contraction via tyrosine kinase activation.
Valero et al. (2006) [197]	1–100 μM	Male Wistar rats	Aorta (denuded)	Relaxation, attenuated by diphenylamine-2-carboxylic acid, glibenclamide, H-89 and bumetanide.
Vera et al. (2007) [230]	10 mg/kg/day administered by gavage for 5 weeks	Female SHRs	Aorta	Potentiation of Ach-mediated vasorelaxation. Attenuation of angiotensin II-mediated vasoconstriction. No effect on NE-mediated vasoconstriction.
Baluchnejadmojarad et al. (2008) [231]	1 mg/kg/day administered intraperitoneally for 4 weeks	Male albino Wistar rats with streptozotocin-induced diabetes and controls	Aorta	Potentiation of Ach-mediated vasorelaxation, partially attenuated by L-NAME and indomethacin. Attenuation of NE and KCl-mediated contraction of aortic rings, with the endothelium removal abolishing the difference between treated and untreated diabetic rats
Galan-Martinez (2008) [151]	1 to 300 μM	Male adult Wistar rats	Aorta (denuded)	Vasorelaxation of KCl-preconstricted vessels.
Je et al. (2009) [152]	0.03and 0.1 mM	Male Sprague-Dawley rats	Aorta	Relaxation of PE-, phorbol ester-, KCl-, fluoride-, and TXA_2_-preconstricted vessels, regardless of endothelial function. Vasorelaxation was not inhibited by iberiotoxin.
Liu et al. (2007) [214]	10^−6^, 10^−5^, or 10^−4^ M	Cows	Pulmonary Arteries and Veins	Relaxation of KCl-, caffeine- and U46619-preconstricted vessels. Genistein prevented U46619-induced tyrosine phosphorylation of a Rho-GEF, a necessary component for Rho kinase action. This leads to the notion that genistein might have inhibited tyrosine kinase.
Nevala et al. (1998) [148]	10^−6^–10^−4^ M	Female and male Wistar rats were used	Mesenteric arteries (intact and denuded)	Relaxation of NE-, KCl- and CaCl_2_-preconstricted vessels, which was unaffected by L-NAME or indomethacin
Honore et al. (1997) [232]	140 mg administered intravenously	Young adult rhesus monkeys	Coronary arteries	Vasorelaxation in monkeys receiving an isoflavone-deprived diet.
Figtree et al. (2000) [38]	10, 20 and 40 mM	Adult New Zealand white rabbits (both genders)	Coronary arteries	Relaxation of KCl-preconstricted vessels, either intact or denuded. The response was unaffected by L-NAME, indomethacin, glibenclamide, barium, methylene blue or ICI 182,780.
Lee et al. (2003) [206]	0.1–100 μM	Pigs	Coronary arteries	Relaxation of U46619-preconstricted vessels, not affected by bradykinin or A23187 but potentiated by SNP and cromakalim. Endothelium denudation and tyrphostin 23 did not affect SNP-induced relaxation.
Kimoto et al. (2005) [207]	10^−6^ M	Pigs	Coronary arteries (denuded)	Mexiletine and lidocaine partially abolished the vasorelaxant response to levcromakalim (i.e. K_ATP_ channel activator). Genistein restored the levcromakalin-induced vasorelaxation in the presence of mexiletine but not of lidocaine.
Ng et al. (2008) [183]	10^−6.5^–10^−4^ M	Pigs	Coronary arteries	Increase in the activity of protein kinase A at high concentration (10^−4.5^ M). At a lower concentration (10^−5.5^ M) it failed to increase PKA activity, unless together with forskolin. SQ22536 (i.e., adenylyl cyclase inhibitor) blocked the genistein-mediated potentiation of PKA, unlike NF 449 (i.e. P2X1 receptor antagonist).
Pinna et al. (2019) [157]	1 nM–0.1 mM	Human subjects	Umbilical veins	Relaxation of 5-HT-preconstricted intact vessels, attenuated by L-NAME, and completely abolished by L-NAME and glibenclamide.
Kitazono et al. (1998) [202]	10^−6^ or 3×10^−6^ M	Male Sprague-Dawley rats	Basilar artery	Attenuation of Ach and bradykinin-mediated vasorelaxation, whereas SNP-mediated relaxation was not affected.
Kitayama et al. (2002) [218]	1 mg/kg chow for 2 months	Male SHR	Basilar artery	Potentiation of Ach- and Y-27632-mediated vasorelaxation in SHR. No attenuation of the increased Rho kinase activity.

**Table 3:** Description and main results of in vitro studies on the vasorelaxant activity of daidzein.

Authors	Compound Concentration	Species and Strain	Type of Vessel	Main Results
Mishra et al. (2000) [149]	10 and 100 μM	Adult male Wistar rats	Aorta	Relaxation of PE-preconstricted vessels, attenuated by endothelium denudation and by L-NAME.
Woodman et al. (2004) [188]	0.2 mg/kg/day administered subcutaneously for 7 days	Male Sprague–Dawley rats	Aorta	Potentiation of Ach-induced vasorelaxation, attenuated by L-NOLA, PPOH, barium, and oubain
Ajay et al. (2003) [150]	0.3 mM	Male Wistar-Kyoto rats	Aorta (intact)	Relaxation of PE and KCl-preconstricted vessels, reduced by L-NAME and indomethacin
Je et al. (2009) [152]	0.01, 0.03 and 0.1 mM	Male Sprague-Dawley rats	Aorta	Relaxation of PE-, KCl-, fluoride-, and phorbol ester-preconstricted vessels
Sharma et al. (2012) [162]	0.2, 0.4 or 0.6 mg/kg/day administered subcutaneously for 7 days	Female Sprague-Dawley ovariectomized rats	Aorta	Potentiation of Ach-, but not of SNP-induced, vasorelaxation. Increased expression of eNOS, calmodulin, and decreased expression of caveolin-1, which prevented ovariectomy-induced vascular dysfunction.
Roghani et al. (2013) [158]	5 or 10 mg/kg administrated by gavage for 7 weeks	Healthy and streptozotocin-induced diabetic Male Wistar rats	Aorta	Relaxation of PE-preconstricted vessels, abolished by endothelium denudation. In treated animals, Ach-induced vasorelaxation was also higher, but L-NAME and indomethacin attenuated this response.
Prawez et al. (2015) [159]	0.5 mg/kg administered subcutaneously for 6 weeks	L-NAME hypertensive male Wistar-rats	Aorta	Relaxation of KCl-preconstricted vessels. Potentiation of Ach-induced vasorelaxation. Decreased potency of SNP-induced vasorelaxation.
Sobey et al. (2004) [161]	0.2 mg/kg/day administered subcutaneously for 7 days	Male Sprague-Dawley rats	Carotid and basilar arteries	Potentiation of Ach-induced vasorelaxation. Potentiation of L-NNA-induced contraction. Expression of caveolin-1 decreased, expression of calmodulin increased and expression of eNOS was unaffected.
Jackman et al. (2007) [99]	10^−7^–10^−3^ M	Adult Sprague–Dawley rats (both genders)	Carotid and basilar arteries	Relaxation of carotid arteries and vasodilation (in vivo) of basilar arteries.
Zhang et al. (2010) [189]	10^−7^–10^−4^ M	Male Sprague–Dawley rats	Basilar artery	Vasorelaxation, inhibited by paxilline, but enhanced by NS1619
Torregrosa et al. (2003) [155]	10^−7^–10^−4^ M	New Zealand White male rabbits	Basilar arteries	Relaxation of KCl or UTP-precontracted vessels, unaffected by endothelial denudation or by L-NAME, ODQ, or NS2028
Nevala et al. (1998) [148]	10^−6^–10^−4^ M	Wistar rats (both genders)	Mesenteric arteries	Relaxation of NE, KCl or CaCl_2_ precontracted vessels, unaffected by endothelium denudation, indomethacin or L-NAME
Nevala et al. (2001) [190]	10–100 mM	Female Wistar rats	Mesenteric arteries (denuded)	Relaxation of NE- and KCl-preconstricted vessels, attenuated by iberiotoxin, charybdotoxin, and apamin

**Table 4:** Description and main results of in vitro studies on the vasorelaxant activity of formononetin.

Authors	Compound Concentration	Species and Strain	Type of Vessel	Main Results
Wu et al. (2010) [167]	10 μM, 100 μM, and 1 mM	Male Sprague-Dawley rats	Aorta	Relaxation of intact vessels, suppressed by L-NAME and methylene blue but not by 1400 W or propranolol. In endothelium-denuded vessels, vasorelaxation was attenuated by methylene blue, glibenclamide, or iberiotoxin. Enhancement of eNOS expression and activity.
Zhao et al. (2012) [184]	10–100 μM	Male Sprague-Dawley rats	Aorta	Relaxation of KCl- and NE-preconstricted vessels
Li et al. (2018) [169]	10^−8^–10^−3^ M	Sprague-Dawley rats	Aorta (intact and denuded)	Relaxation of KCl-preconstricted vessels, suppressed by endothelium denudation and by L-NAME
Sun et al. (2011) [154]	1–300 μM	Sprague-Dawley rats	Mesenteric, renal, basilar, coronary and aortic arteries (intact and denuded)	Relaxation of PE- or U46619-preconstricted vessels, attenuated by endothelium denudation
		Male SHRs	Mesenteric arteries	Relaxation of PE- or U46619-preconstricted arteries, attenuated by endothelium denudation and by L-NAME but unaffected by indomethacin or glibenclamide
Sun et al. (2016) [170]	1–10 μM	Male Sprague-Dawley rats	Mesenteric arteries	Vasorelaxation, attenuated by ICI 182780. Expression of eNOS increased via ERK and JNK activation.
Tseng et al. (2016) [168]	1 nM–100 μM	Male Sprague-Dawleyrats	Mesenteric arteries	Relaxation of intact vessels, suppressed by L-NAME and glibenclamide
Sun et al. (2013) [225]	50 mg/kg administered orally for 8 weeks	Male SHRs	Mesenteric arteries	Attenuation of NE or 5-HT-induced vasoconstriction. Potentiation of Ach-induced vasodilation. Expression of alpha-1 adrenergic and 5-HT_2A/1B_ receptors in VSM cells decreased.

**Table 5:** Description and main results of in vitro studies on the vasorelaxant activity of biochanin A.

Authors	Compound Concentration	Species and Strain	Type of Vessel	Main Results
Choi et al. (2014) [176]	10^−7^–10^−4^ M	Male Sprague-Dawley rats subjected to 2K1C-induced renovascular hypertension	Aorta	Endothelium-dependent vasorelaxation was attenuated by endothelium denudation, by glibenclamide, TEA and by 4-aminopyridine
Wang et al. (2005) [185]	10^−9^–10^−4^ M	Sprague-Dawley rats	Aorta	Relaxation of PE-, KCl-, and CaCl_2_-preconstricted vessels, intact and endothelium-denuded, attenuated by TEA and glibenclamide but not by 4-aminopyridine
Wang et al. (2006) [175]	10^−7^–3×10^−4^ M	SHRs	Aorta	Vasorelaxation, attenuated by endothelium denudation, by glibenclamide and 4-aminopyridine but not by L-NAME or indomethacin
Migko et al. (2020) [177]	10^−7^–10^−3^ M	Wistar Han rats	Aorta	Relaxation of NE-preconstricted vessels.
	3×10^−6^–3×10^−5^ M	Pigs	Coronary arteries	Relaxation of KCl-, CaCl_2_-, 5-HT-, and U46619-preconstricted vessels. Endothelium denudation did not affect relaxation of U46619-preconstricted vessels. Potentiation of vasorelaxation by SNP but not by forskolin.
Torregrosa et al. (2003) [155]	10^−6^–10^−4^ M	New Zealand White male rabbits	Basilar artery	Vasorelaxation, unaffected by endothelium denudation, by L-NAME, indomethacin, ODQ, or NS2028
Figtree et al. (2000) [38]	3, 10 and 30 μM	Adult male or non-pregnant female New Zealand white rabbits	Coronary arteries	Relaxation, independent of gender, of intact and denuded vessels

**Table 6:** Description and main results of in vitro studies on the vasorelaxant activity of equol and other metabolites.

Authors	Compound Concentration	Species and Strain	Type of Vessel	Main Results
Chin-Dusting et al. (2001) [178]	Dihydrodaidzein, cis-, and trans-tetrahydrodaidzein, dehydroequol (1 μg/mL)	Male Sprague-Dawley rats	Aorta	Vasorelaxation, inhibited by endothelium denudation, by L-NOLA, ODQ, or KCl
Joy et al. (2006) [179]	Equol (0.03–15 μM)	Sprague-Dawley rats	Aorta	Vasorelaxation by stimulation of NO release, a response that is unaffected by ICI 182,780 or pertussis toxin
Ohkura et al. (2015) [180]	Equol (200 mg/day for 5 weeks via osmotic pump)	Isoflavone-deficient ovariectomized Sprague-Dawley rats	Carotid arteries	Restoration of Ach-mediated vasorelaxation, which was suppressed by N^ω^-monomethyl-L-arginine acetate (L-NMMA)
Kim et al. (2015) [181]	Equol (10^−11^–10^−6^ M)	Human subjects	Uterine arteries	Relaxation of PE-, but not of KCl-preconstricted vessels
Cheng et al. (2010) [182]	Equol (0.1, 1, and 10 μM)	Pigs	Pulmonary arteries	Restoration of ritonavir-induced reduction in eNOS expression. Restoration of bradykinin-mediated vasorelaxation.

## 4. Effect on Blood Pressure and Blood Flow

Studies conducted both in animals and in humans have shown that isoflavones are able to reduce blood pressure by modulating several cardiovascular activities. Apart from their vasorelaxant activity, isoflavones are also able to modulate baroreceptor sensitivity, catecholamine synthesis at the adrenal medulla, as well as to affect the renin–angiotensin–aldosterone (RAA) endocrine axis. Most studies published thus far, however, have assessed the effect of mixtures of isoflavones, which hinders the knowledge of which bioactive compounds may be responsible for the observed beneficial effects and whether those effects are cumulative or not. Besides the chemical heterogeneity of soy-based products, several other factors contribute to the variability in terms of results, such as study type and duration, inclusion and non-inclusion criteria, source of isoflavones (foodstuffs, extracts), and the profile of intestinal metabolism (equol producers, non-producers). Several systematic reviews and meta-analyses have been conducted, hoping to shed light on the effects of isoflavone supplementation on blood pressure, but only a few studies so far have focused on the effects of isolated compounds. Table 7 summarizes the main findings of the studies that assessed the blood pressure lowering effects of isolated isoflavones in animals and in humans.

In stroke-prone male SHRs, the chronic administration of genistein (0.6 mg/g diet for 7 weeks) attenuated the increase of blood pressure caused by dietary sodium chloride, a response blunted by hexamethonium (i.e., ganglion blocker). During this intervention, an increase in NO plasma levels was also noted. These results suggest that the hypotensive effect of genistein is due to an increased production of NO at the vascular endothelium and/or due to the modulation of an autonomic nervous system response [233]. In ovariectomized SHRs, the chronic administration of genistein (10 mg/kg/day by gavage for 5 weeks) reduces the systolic blood pressure [230]. When administered intravenously to the brachial artery of healthy human subjects, genistein increases forearm blood flow with a similar potency to that of 17β-estradiol. It also potentiated the vasodilation response to Ach but not to SNP, and its effect was attenuated by N^ω^-monomethyl-L-arginine acetate (L-NMMA, i.e., NOS inhibitor), suggesting again that genistein increased the endothelial secretion of NO [234]. In post-menopausal women, the chronic ingestion of genistein (54 mg/day for 1 year) significantly improves flow-mediated dilation of the brachial artery with a similar potency to an estrogen/progestin scheme, in addition to lowering the plasma endothelin-1 levels [235,236].

In L-NAME-induced hypertensive rats, administration of daidzein (0.5 mg/kg subcutaneous) for 6 weeks reduces the mean blood pressure in comparison to untreated animals. Moreover, thoracic aortae from daidzein-treated rats showed higher dilation to Ach in comparison to untreated animals [159]. Daidzein sulfates show a hypotensive effect in SHR at different concentrations (20 mg/kg and 40 mg/kg), with single or multiple (1/day for 2 weeks) oral administrations. Daidzein (20 mg/kg) decreases blood pressure in multidose (more slowly than for sulfates) but not in single dose administrations [237]. In a study performed in pre-hypertensive and untreated hypertensive, post-menopausal and equol-producing female subjects, daidzein (63 mg) taken orally for 6 months neither changed their blood pressure nor improved their flow-mediated dilation [98]. When daidzein is administered intravenously to the brachial artery of healthy young subjects, it fails to change forearm blood flow [234].

The chronic administration of formononetin (80 mg/day for 5 weeks) to normotensive human subjects does not change blood pressure or improve flow-mediated dilation [12]. When formulated in enteric-coated microparticles and administered to hypertensive ovariectomized rats, biochanin A (10 mg/kg, p.o. for 1 week) significantly decreases the systolic, diastolic, and mean blood pressure [238]. This effect seems to be largely attributed to an endothelium-dependent decrease in peripheral resistance, as co-administration with L-NAME prevented this hypotensive effect [238]. When administered to SHRs for 8 weeks, formononetin (50 mg/kg/day) lowers blood pressure and shows endothelium-dependent vasodilation [225]. The chronic administration of biochanin A (80 mg/day for 5 weeks) to normotensive human subjects does not change the blood pressure nor improve the flow-mediated dilation [12].

The effect of several isoflavone metabolites on blood pressure and perfusion was also examined. The topical administration of equol to surgically exposed basilar arteries in Sprague-Dawley rats results in a weak vasodilation and antioxidant effect. Presumably, equol might react with reactive oxygen species (ROS) and prevent a decline in endothelial NO levels [99]. In deoxycorticosterone acetate/salt-induced hypertensive rats, the chronic administration of equol (10 and 20 mg/kg for 5 weeks) reduces their blood pressure, presumably by increasing endothelial NO synthesis and inhibiting RAA axis [239]. When dehydroequol is administered intravenously to the brachial artery in healthy human males, forearm blood flow increases, a response that is blunted by L-NMMA [240]. The effect of trans-tetrahydrodaidzein (i.e., a metabolite of daidzein and formononetin) was examined in humans. In middle-aged subjects with significant cardiovascular risk (overweight, dyslipidemia, and impaired glucose metabolism), the oral supplementation with trans-tetrahydrodaidzein (1 g/day for 5 weeks) significantly reduced blood pressure and arterial stiffness [241].

In humans consuming soy-free diets, the plasma concentration of isoflavones lies in the nanomolar range, typically under 40 nM [242]. The ingestion of dietary soy increases the plasma concentration up to the micromolar range (7–8 μM), for example in East Asian populations and in vegetarians, as does the ingestion of soy-based foodstuffs and supplements [21,22,23]. One study in healthy subjects has shown that, after the ingestion of a commercially available soy protein drink containing 37 mg genistein, the plasma concentration of genistein reaches levels of approximately 2 mM [38]. Given that the vasorelaxation effects attributed to isoflavones are mostly obtained with plasma concentrations in the micromolar range, it becomes apparent that isolated isoflavones should only exert vasodilation in vivo when supplemented in concentrated drinks/beverages. In contrast, when absorbed through long-term dietary intake, these beneficial vascular effects are probably attributed to a synergistic action of different isoflavones in addition to a possible modulation of the intestinal isoflavone metabolism by intestinal flora.

### 4.1. Effect on the Renin–Angiotensin–Aldosterone Axis

The RAA axis consists of an endocrine system that contributes to blood pressure regulation by modulating peripheral vascular resistance. When kidney perfusion pressure decreases, the kidney granular cells increase the secretion of the enzyme renin. In the bloodstream renin converts the liver peptide angiotensinogen to angiotensin I; both of these are biologically inactive. Then, angiotensin I is converted to angiotensin II by ACE, an enzyme expressed in the endothelial cells of pulmonary and renal circulations. Angiotensin II can be converted to angiotensin-(1–7) by ACE2 and to angiotensin III by aminopeptidase A (APA). Finally, angiotensin III is converted to angiotensin IV by aminopeptidase D. The several mediators of this pathway exert many different functions in the organism. At the vascular level, angiotensin II and angiotensin III are vasoconstrictor mediators, whereas angiotensin-(1–7) and angiotensin IV produce vasodilation [243].

Several isoflavones interfere with the RAA axis. Genistein inhibits the expression of ACE in a concentration-dependent way in the serum of Wistar [244] as well as in the serum, aorta, and aortic endothelial cells of Sprague-Dawley rats [213]. In the latter, this response is blocked by tamoxifen (i.e., nonspecific estrogen receptor antagonist), showing that this effect is mediated by the estrogen receptor. Moreover, the compound PD98059 (i.e., ERK1/2 phosphorylation inhibitor) abolished this response, also showing that genistein inhibits ACE through activation of the ERK1/2 pathway [213]. When incubated with aortic endothelial cells from stroke-prone SHRs, genistein suppressed the expression of the angiotensin II type 1 receptor in a concentration- and time-dependent manner [245]. In different animal models genistein has produced different results regarding its capacity to modulate the RAA axis in vivo. When administered in the diet (0.6 mg/g diet for 7 weeks) of stroke-prone SHRs, it failed to significantly change the levels of renin and aldosterone [233]. However, a two-day pretreatment with genistein (25 mg/kg intravenously) prevents the hypertensive response of angiotensin I, accentuates the hypotensive response of bradykinin, but does not alter the hypertensive effect of angiotensin II [244]. Similarly, when administered intravenously to conscious Sprague-Dawley rats, it fails to suppress the hypertensive response to angiotensin II [246]. Daidzein is known to inhibit ACE activity in vitro and to display significant hypotensive effects in vivo. The intravenous administration of daidzein (25 mg/kg) attenuates the increase in blood pressure induced by angiotensin I in healthy Wistar rats [247]. Daidzein inhibits ACE activity in vitro in a concentration-dependent manner. Rat plasma samples treated with daidzein (3–300 μmol/L) show significantly lower ACE activity [247]. Similar concentrations were previously found to inhibit ACE activity in rat aortic endothelial cells [213]. Formononetin does not seem to affect the RAA axis in vivo. When administered chronically (50 mg/kg/day for 8 weeks), it did not significantly change the serum concentrations of renin, angiotensin II, and aldosterone [225]. In deoxycorticosterone acetate salt-induced hypertensive rats, the chronic administration of equol (10 and 20 mg/kg for 5 weeks) decreased ACE activity and expression, increased ACE2 expression, and decreased plasma angiotensin I and aldosterone, thus showing an inhibitory activity on the RAA axis with a potency equivalent to that of lisinopril (i.e., ACE inhibitor) [239].

### 4.2. Effect on Baroreceptor Sensitivity

Isoflavones are also able to interfere with the blood pressure-regulating mechanisms. To date, the only isoflavone to be able to modulate baroreceptor sensitivity was genistein. At concentrations of 50–200 μmol/L, genistein inhibits the carotid baroreceptor in male anesthetized Sprague-Dawley rats. This inhibition is not blocked by L-NAME but rather by Bay K8644 or by sodium orthovanadate (i.e., inhibitor of tyrosine phosphatase). This suggests that genistein inhibits the baroreceptor by inhibiting tyrosine kinase and inducing vasorelaxation as well as by blocking calcium influx into the baroreceptor neurons [248]. Besides acting peripherally to modulate baroreceptor sensitivity, the topical application of genistein on the medulla of anesthetized Sprague-Dawley rats reduces PE-induced reflex bradycardia [249].

### 4.3. Effect on Catecholamine Synthesis

Genistein and daidzein are known to affect catecholamine synthesis. Genistein (0.1–10 μM) is able to increase the uptake of noradrenaline into SK-N-SH and COS-7 cells [250]. Daidzein seems to display a dual effect on catecholamine secretion. At low concentrations (10–100 nM), daidzein stimulates catecholamine synthesis on bovine medullary cells by increasing the tyrosine hydroxylase activity. At high concentrations (≥1 μM), however, daidzein inhibits catecholamine synthesis [251].

### 4.4. Possible Connection between Effects on Immunity and Blood Pressure

Isoflavones are also known to affect immune function in a variety of ways. They are able to modulate the numbers of circulating immune cells, such as eosinophils and lymphocytes as well as the levels of lymphocytes in the thymus and spleen. They can also modulate the maturation and reactivity of immune cells, such as natural killer, T lymphocyte, macrophage, and dendritic cells, as well as modulate cytokine production [252]. Although there are currently no studies providing a direct connection between the immune modulation activity of isoflavones and blood pressure regulation, it is possible that such an activity may directly and indirectly affect vascular tone, vascular permeability, and even changes in vascular histology.

**Table 7:** Description and main results of in vivo studies on the blood pressure-lowering effects of isolated isoflavones or isoflavone-containing products (SBP—systolic blood pressure, DBP—diastolic blood pressure, MBP—mean blood pressure).

Authors	Compound Concentration/Dosage and Duration of Treatment	Animal Species and Strain	Blood Pressure Measurement Technique	Main Results
Vera et al. (2007) [230]	Genistein (10 mg/kg/day) by gavage for 5 weeks	Ovariectomized SHRs	Tail-cuff plethysmography	Reduction of SBP after 5 weeks
Prawez et al. (2015) [159]	Daidzein (0.5 mg/kg) administered subcutaneously for 6 weeks	L-NAME-induced hypertensive rats	Invasive pressure transducer	Reduction of MBP in comparison to untreated animals
Cao et al. (2006) [237]	Daidzein sulfates (20 mg/kg and 40 mg/kg) with a single or multiple (1/day for 2 weeks) oral administrations	SHRs	Tail-cuff plethysmography	Blood pressure reduction with single or multiple administrations. Daidzein decreases blood pressure in multidose (more slowly than for sulfates) but not in single dose administrations
Liu et al. (2015) [98]	Daidzein (63 mg) taken orally for 6 months	Pre-hypertensive and untreated hypertensive post-menopausal subjects	Portable device, calibrated by a mercury sphygmomanometer	No change in blood pressure. No improvement in flow-mediated dilation was observed.
Sachdeva et al. (2016) [238]	Biochanin A (10 mg/kg, p.o. for 1 week)	Hypertensive ovariectomized rats	Tail-cuff plethysmography	Reduction of SBP, DBP and MBP, blunted by L-NAME
Sun et al. (2013) [225]	Formononetin (50 mg/kg/day for 8 weeks)	SHRs	Tail-cuff plethysmography	Blood pressure reduction and endothelium-dependent vasodilation
Palanisamy and Venkataraman (2013) [253]	Genistein (1 mg/kg/day for 44 days) by oral administration	Fructose-fed hypertensive male Wistar rats	Tail-cuff plethysmography	No significant change in blood pressure

## 5. Conclusions

Isoflavones are phytoestrogen compounds with recognized beneficial effects in the cardiovascular system. Owing to minor structural differences, isoflavones modulate many cellular pathways that culminate in vasorelaxation, acting either in the endothelium, vascular smooth muscle, or both. Dietary intake or supplementation allow the attainment of sufficiently high plasma concentrations to produce significant vasodilation and lower blood pressure. Most studies have assessed the blood-pressure-lowering potential of these compounds in subjects consuming mixtures of isoflavones, with conflicting results, probably due to confounding variables. Only a few studies have focused on isolated compounds, where promising results were found, which should prompt future clinical studies. In order to better understand the cardiovascular potential of isoflavones, future studies should take into account not only the ingested dose of isoflavones but also the subjects’ dietary habits and medical history, as well as the role of intestinal flora. This paper contributes to highlight isolated isoflavones as potentially suitable alternatives to soy-based foodstuffs and supplements, which could enlarge the current therapeutic arsenal.

## Figures and Tables

**Table 1:** Description and main results of the clinical studies on the blood pressure-lowering effects of isoflavones (SBP – systolic blood pressure, DBP – diastolic blood pressure).

Authors	Study Type (Number of Studies Considered)	Isoflavone-Containing Product	Main Results
Hooper et al. (2008) [46]	Systematic review and meta-analysis of randomized clinical trials (*n* = 83)	Soybeans, soy protein isolate, and isoflavone extracts	Significant decrease in DBP
Arenas et al. (2008) [47]	Systematic review and meta-analysis of observation studies and clinical trials (*n* = 14)	Soy products	No significant variations in SBP or DBP between subjects treated with isoflavones and non-treated subjects
Taku et al. (2010) [48]	Systematic review and meta-analysis of randomized clinical trials (*n* = 14)	Isoflavones extract	Significant decrease in SBP in normotensive and in pre-hypertensive patients, with greater effects in interventions longer than 3 months
Dong et al. (2011) [49]	Systematic review and meta-analysis of randomized clinical trials (*n* = 27)	Soy protein containing isoflavones	Significant decrease in SBP and DBP in normotensive and in hypertensive subjects, more markedly in the latter. Blood pressure reductions were related to the pre-treatment BP levels of the subjects and the type of control diet used as comparison
Liu et al. (2011) [50]	Systematic review and meta-analysis of randomized clinical trials (*n* = 11)	Soy protein containing isoflavones	Significant decrease in SBP and DBP in hypertensive subjects (*n* = 5 trials) but not in normotensive subjects (*n* = 6 trials)
Yan et al. (2017) [51]	Systematic review and meta-analysis of observational studies (*n* = 17)	Soy products	Significant negative correlation between soy intake and the risk of cardiovascular disease, stroke, and coronary heart disease
Namazi et al. (2018) [52]	Systematic review and meta-analysis of cohort studies (*n* = 7)	Soy products	No significant association between high consumption of soy products and lower risk of mortality from cardiovascular disease
Nachvak et al. (2019) [53]	Systematic review and meta-analysis of prospective studies (*n* = 24)	Soybeans and soy products	Inverse relationship between consumption of soy products and cardiovascular mortality
Li et al. (2020) [54]	Umbrella review of epidemiological and clinical studies (*n* = 114)	Soybeans and soy products	Generally, isoflavone consumption is more beneficial than detrimental. A beneficial role in cardiovascular disease was observed

## Data Availability

No new data were created or analyzed in this study. Data sharing is not applicable to this article.
